# Surgical helmet systems in total joint arthroplasty: assessment of hood sterility and donning technique

**DOI:** 10.1186/s42836-023-00212-4

**Published:** 2023-11-15

**Authors:** Timothy McAleese, Tiarnán Ó Doinn, James M. Broderick, Ross Farrington, Anna-Rose Prior, John F. Quinlan

**Affiliations:** 1 RCSI University of Medicine and Health Sciences, Dublin, D02 YN77 Ireland; 2 Department of Trauma and Orthopaedics, Tallaght University Hospital, Dublin, D24 NR04 Ireland; 3 Department of Microbiology, Tallaght University Hospital, Dublin, D24 NR04 Ireland

**Keywords:** Surgical helmet systems, Arthroplasty, Periprosthetic joint infection, Donning, Sterile surgical field

## Abstract

**Background:**

The incidence of prosthetic joint infection (PJI) is increasing, coincident with the rising volume of joint arthroplasty being performed. With recent controversy regarding the efficacy of surgical helmet systems (SHS) in preventing infection, the focus has turned to the correct donning techniques and usage of surgical hoods. The aim of this study was to compare the bacterial contamination of the operating surgeon’s gloves after two common donning techniques of SHS hoods. We also evaluated the baseline sterility of the SHS hoods at the beginning of the procedure.

**Methods:**

The bacterial contamination rate was quantified using colony-forming units (CFUs), with 50 trials performed per donning technique. Samples were cultured on 5% Columbia blood agar in ambient air at 37 °C for 48 h and all subsequent bacterial growth was identified using a MALDI-TOF mass spectrometer. In Group 1, the operating surgeon donned their colleague’s hood. In Group 2, the operating surgeon had their hood applied by a non-scrubbed colleague. After each trial, the operating surgeon immediately inoculated their gloves onto an agar plate. The immediate sterility of 50 SHS hoods was assessed at two separate zones—the screen (Zone 1) and the neckline (Zone 2).

**Results:**

There was no significant difference in contamination rates between the two techniques (3% vs. 2%, *P* = 0.99) or between right and left glove contamination rates. Immediately after donning, 6/50 (12%) of SHS hoods cultured an organism. Contamination rates at both the face shield and neckline zones were equivalent. The majority of bacteria cultured were Bacillus species.

**Discussion:**

We found no significant difference in the operating surgeon’s glove contamination using two common SHS hood-donning techniques when they were performed under laminar airflow with late fan activation. We suggest the SHS hood should not be assumed to be completely sterile and that gloves are changed if it is touched intraoperatively.

## Background

International registry data provides evidence that the incidence of prosthetic joint infection (PJI) is increasing [1, 2]. This has largely been attributed to an exponential rise in the volume of primary arthroplasty being performed each year in our aging population [3]. Combined data from multiple national registries suggests that the current mean rates of PJI for total hip arthroplasty (THA) and total knee arthroplasty (TKA) are 0.97% and 1.03%, respectively [4].

PJI is a devastating complication and a leading cause of morbidity, mortality as well as healthcare-related costs. Bacteria shed from operating room personnel have long been recognized as a source of intraoperative contamination and reducing this transmission remains a focus for quality improvement efforts [5–8]. Commonly used interventions include laminar airflow, surgical caps, facemasks, sterile gowns and body exhaust systems (BES) or surgical helmet systems (SHS). The BES was pioneered by Sir John Charnley in the 1960s and used a closed “negative pressure” system to successfully prevent airborne transmission of bacteria [9]. Modern “positive pressure” SHS or “space suits” remain contentious with conflicting evidence regarding their ability to lower deep infection rates [10–13]. Nonetheless, SHS are commonly used during arthroplasty surgery if not only for their value as personal protective equipment.

Continued efforts to identify potential sources of intraoperative infection have increasingly focused on operating room attire and their methods of application [10, 14]. Research has been conducted into sterile glove donning techniques (closed vs. open vs. staff-assisted) [15, 16], methods for opening sterile glove packaging (direct hand off vs. direct drop) [17] as well as gowning techniques (self-gowning vs. assisted-gowning) [18].

Hospital practices can differ considerably regarding how SHS hoods are used and there are a variety of donning techniques. In some facilities, a non-sterile team member applies the hood over the surgeon in an attempt to keep the surgeon’s hands and SHS as sterile as possible. In other hospitals, surgical team members assist each other with donning the SHS. This poses a risk of glove contamination from contact with the unsterile, plastic helmet underneath or from contact with their colleague’s shoulders. Similarly, donning one’s own hood without direct visualization risks contamination from the unsterile helmet or shoulders.

There is currently no consensus on the appropriate method of donning surgical hoods. Therefore, the aim of this study was to compare the bacterial contamination of the operating surgeon’s gloves using two common SHS donning techniques. Our secondary aim was to determine the baseline sterility of the SHS hood at the beginning of a procedure.

## Methods

### Equipment and study design

Two techniques of hood donning were compared in this experimental study. Fifty surgical scrubs were performed for each donning method, resulting in a total of 100 trials. Ethical review board approval was not required to undertake this study as it did not involve human subjects.

**Fig. 1:**
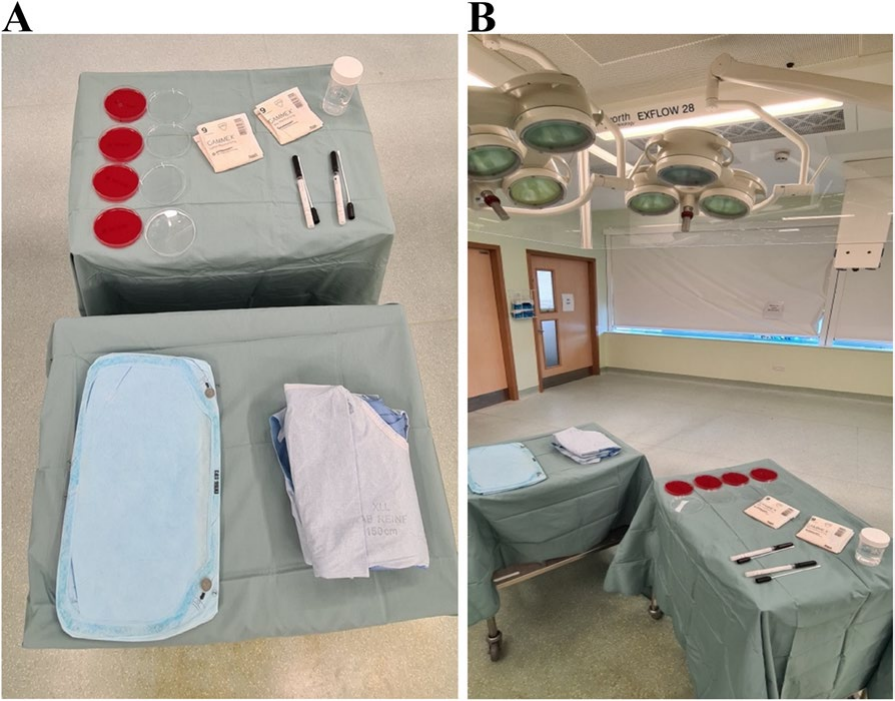
**A**, **B** The materials and setting of agar plate inoculation for this study. This was undertaken by exercising strict sterile precautions under laminar airflow with late fan activation. Agar plates were only opened immediately before inoculation. Pre-moistened swabs were used to collect samples from the SHS hoods

All trials were conducted using a strict sterile technique in an operating theatre and all donning took place under HEPA-filtered, vertical laminar airflow (Howorth Exflow 28). A sterile gown (3 M™ Basic Surgical Gown; 3 M Ireland, Dublin, Ireland), two sterile gloves (Gammex latex gloves; Ansell Healthcare products, Brussels, Belgium) and a disposable sterile surgical helmet system (SHS) hood (Stryker Flyte^®^ Sterishield^®^ personal protection system; Stryker Medical, New Jersey, USA) were laid out on a sterile trolley under laminar airflow. The SHS comprised an unsterile helmet with an in-built fan for ventilation covered with a sterile hood. The unsterile helmet was worn before scrubbing but the fan was not activated until after the hood was applied. Two orthopaedic registrars simulated the role of a surgeon and surgical assistant (Fig. 1A, B).

**Fig. 2:**
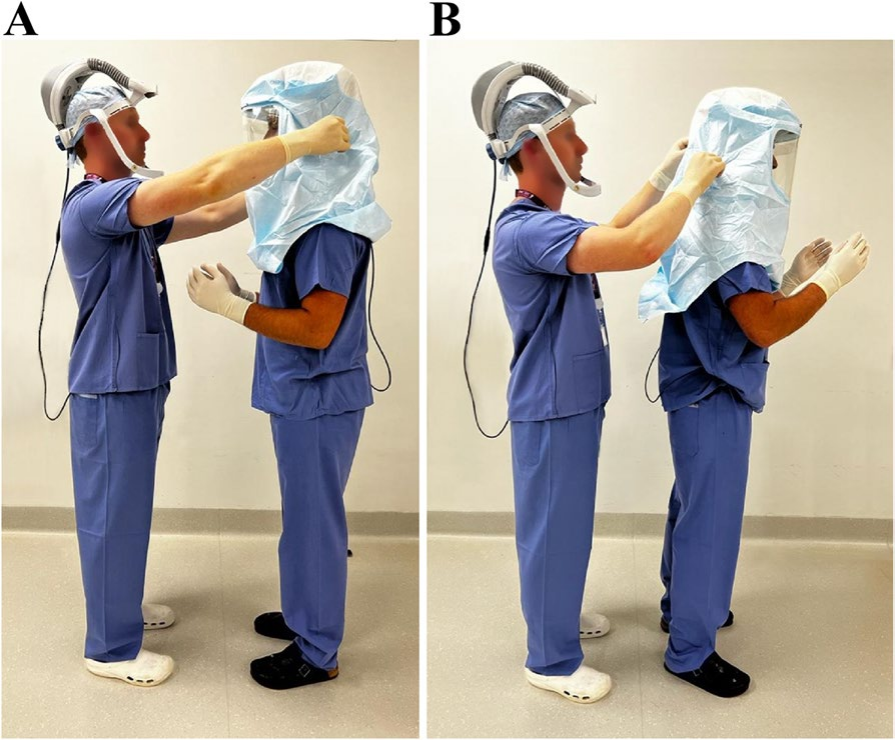
**A**,** B** Group 1 evaluated the rate of contamination of the operating surgeon’s gloves (left) after touching the SHS hood while assisting another surgical team member with donning

In Group 1, the surgeon performed a full surgical scrub using 7.5% (w/w) Povidone-Iodine solution (Videne, Ecolab) before donning sterile gloves. They then placed the SHS hood over their colleague before immediately press-inoculating all 5 fingers of both gloves onto 5% Columbia blood agar. A separate agar plate was used for each hand. This group was used to investigate the rate of contamination of the operating surgeon’s gloves after touching the SHS hood while assisting another surgical team member with donning them (Fig. 2A, B).

**Fig. 3:**
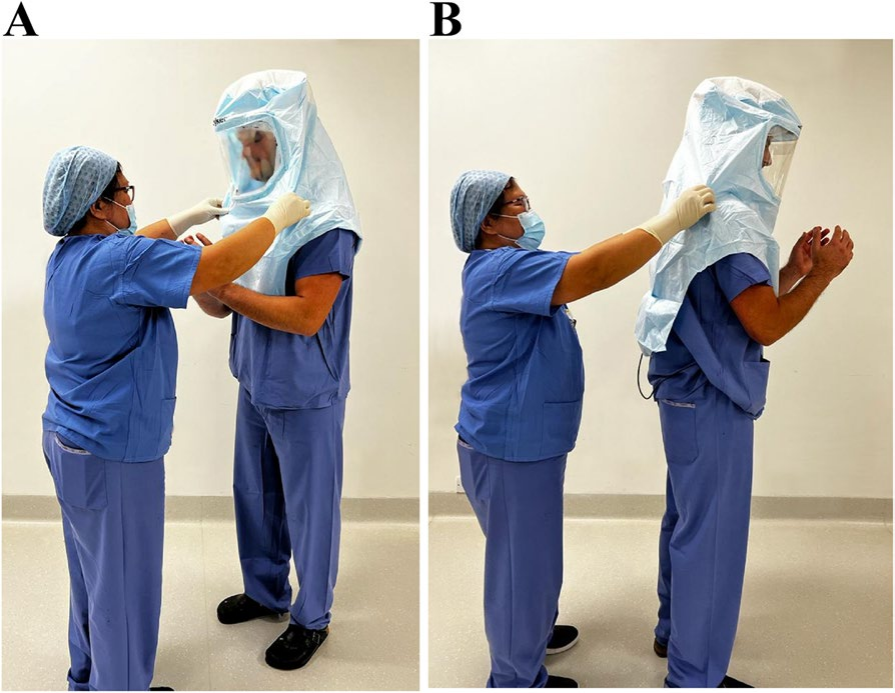
**A**, **B** Group 2 evaluated the rate of contamination of the operating surgeon’s gloves (right) when their hood was donned by a non-scrubbed colleague. This technique avoided touching and manipulating the SHS hood

In Group 2, the surgeon performed a full surgical scrub before a non-scrubbed colleague (wearing sterile gloves) donned the surgeon’s surgical hood. The surgeon then donned their sterile gloves before immediately press-inoculating all 5 fingers of both gloves onto agar. This group was used to investigate the contamination of the operating surgeon’s gloves having never touched or manipulated the SHS hood (Fig. 3A, B).

**Fig. 4:**
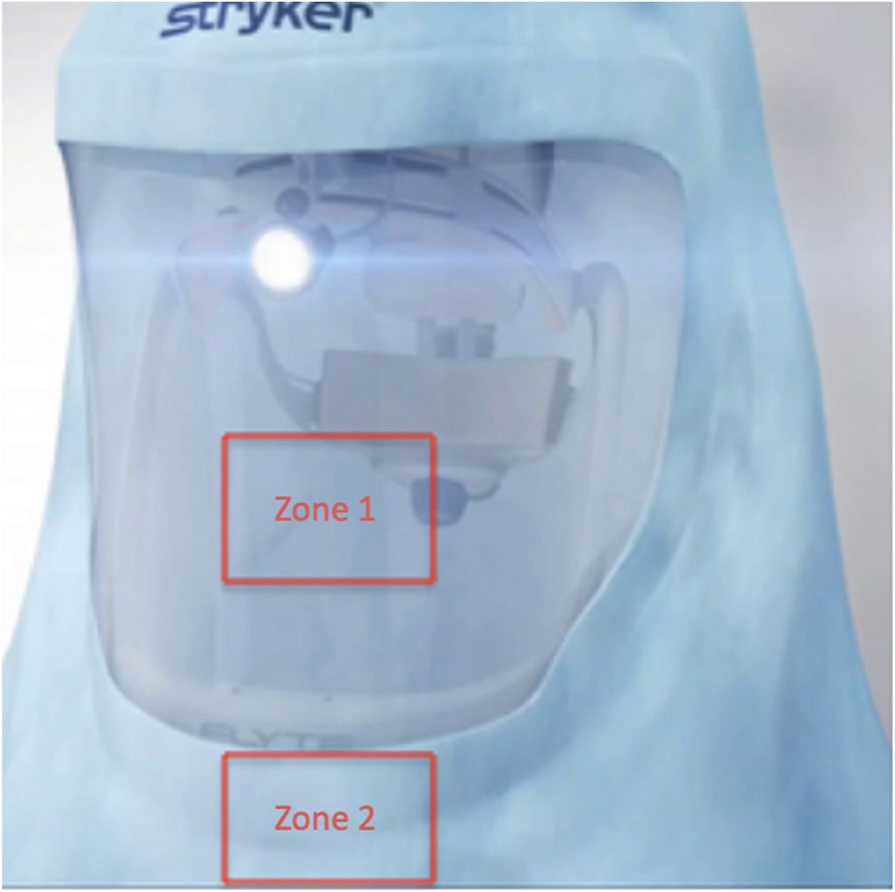
Two 3 cm × 3 cm zones on 50 hoods were swabbed. Firstly, the screen area (Zone 1) was swabbed. This was followed by the neckline (Zone 2) facing the sterile field

The second aim of this study was to determine the baseline sterility of the SHS hoods. Immediately after the hood was placed over the helmet, sterile culture swabs were taken from two separate zones: the screen (Zone 1) and the neckline (Zone 2). This was repeated for 50 hoods (Fig. 4).

### Microbiological testing

Sterile gloves were worn throughout sample collection and swabbing. As part of a well-accepted semi-quantitative technique, Columbia blood agar supplemented with 5% sheep blood was used (Columbia blood agar, Fannin Healthcare, Ireland). This is a non-selective medium known to support the growth of numerous bacterial strains, including Gram-negative and Gram-positive species.

**Fig. 5:**
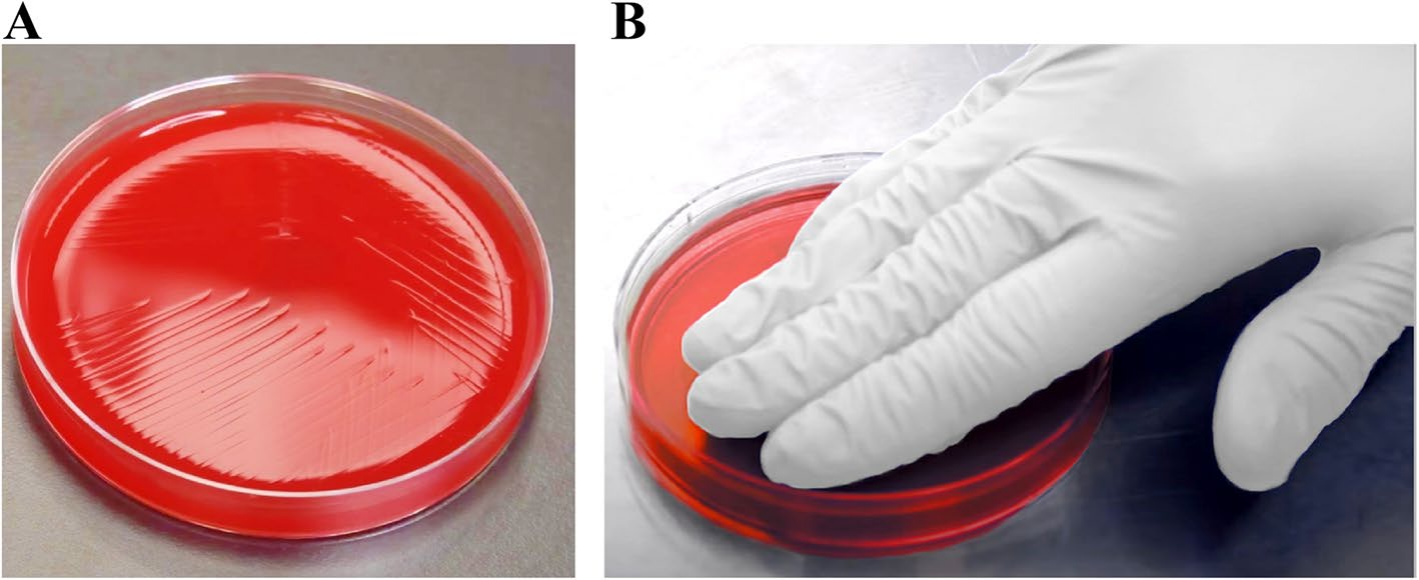
**A**,** B** Columbia agar with 5% sheep blood was inoculated with sterile swabs (Amies gel/charcoal transport medium) using the 4-quadrant streak method. The press-inoculation technique was used to sample all 5 fingers on both sterile gloves

We used the “press-inoculation” technique to sample all 5 fingertips on each glove. A separate agar plate was utilized for each glove to allow for comparison between the right and left hand. Initially, the 4 fingers, excluding the thumb, were pressed in a row against the agar medium and held for 2 s. This was followed by placing the thumb into the remaining space on the agar plate. To sample the two zones on the SHS hood, we used sterile swabs with Amies gel/charcoal transport medium (BD BBL™ CultureSwab Plus™ EZ, Ireland**)**. These were pre-moistened in normal saline to facilitate extraction of any contaminant on the hood surface. Separate sterile swabs were employed to sample each of the 2 zones. Each plate was only opened immediately prior to inoculation. The 4-quadrant streak method was used to inoculate each plate (Fig. 5A, B).

**Fig. 6:**
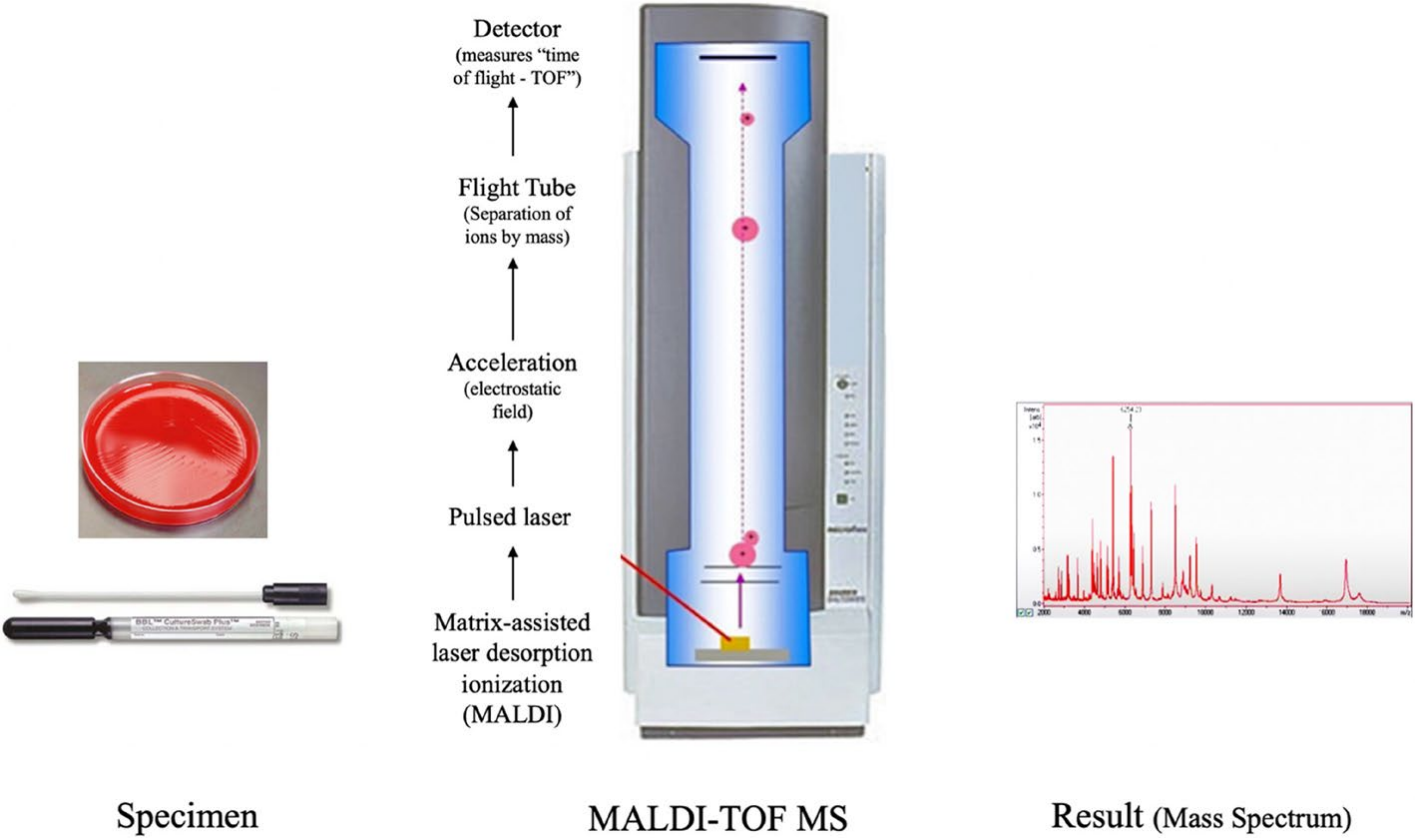
The MALDI-TOF mass spectrometer was used to identify any bacterial growth

Plates were incubated in ambient air at 37 °C for 48 h before they were examined for bacterial growth. Isolated, pure colonies were initially characterized according to the Gram staining results. All positive samples were identified using a MALDI-TOF mass spectrometer (Fig. 6). All hoods were assumed to be sterile before use and therefore, any growth was considered significant and reported, regardless of the number of colony-forming units (CFUs).

### Statistical analysis

Categorical variables are displayed as numbers and percentages. Contingency analysis comparing the presence of contamination between donning methods and hood zones was carried out using McNemar’s test. The threshold for statistical significance was set at a *P*-value < 0.05. Data analysis was performed using SPSS Version 28.

## Results

There was no significant difference in the microbial contamination rates of the operating surgeon’s gloves between the two donning methods (2% in Group 1 vs. 3% in Group 2, *P* = 0.99). In Group 1, where the operating surgeon assisted in donning another surgeon, 98% of gloves were sterile. Two right-hand gloves grew 1 CFU of Bacillus Species and 1 CFU of Rahnella Aquatis, respectively. In Group 2, where the operating surgeon was gowned by a non-scrubbed team member, 97% of gloves were sterile. Coagulase-negative staphylococcus (1 CFU) was cultured on one left-hand glove and 2 right-hand gloves growing 1 CFU and 2 CFUs of Bacillus species, respectively. There was no statistically significant difference in the contamination rate between the right and left hands (Table 1).

**Table 1:** Comparison of the operating surgeon’s glove contamination after two different SHS hood-donning methods

	**Group 1** Operating surgeon donning a scrubbed colleague	**Group 2** Operating surgeon donned by non-scrubbed colleague	*P*-value
**Total no. of agar plates**	100	100	
**Contamination rate**	2%	3%	*P* = 0.99
**Right Hand** **(*n* = 50)**	2 gloves • Bacillus Sp. 1 CFU • Rahnella Aquatis 1 CFU	1 glove • CoNS 1 CFU	
**Left Hand** **(*n* = 50)**	0 gloves	2 gloves • Bacillus Sp. 2 CFU • Bacillus Sp. 1 CFU	

*Sp.* species, *CFU* colony-forming unit

At time zero, immediately after the SHS was donned, 6/50 (12%) of SHS hoods had positive cultures that isolated an organism. Three of the hood screens (Zone 1) cultured Bacillus species. Of note, Hood 11 was completely overgrown with an organism (> 100 CFU) that the MOLDI-TOF failed to identify. This was believed to be a false positive, secondary to contamination of the petri dish. Four of the samples from the hood neckline (Zone 2) isolated bacteria, of which 3/4 (75%) were Bacillus species. Hood 23 cultured an unquantifiable swarming colony and *Paenibaccillus Glucanolyticus* (Table 2).

**Table 2:** Baseline sterility of the SHS hood immediately after donning. 6/50 (12%) of SHS hoods were contaminated with an organism. The bacterial overgrowth of Zone 1 on Hood 11 was agreed to be a contaminant

**Swab of Zone 1** (3 × 3 cm on screen: *n* = 50)	**Swab of Zone 2** (3 × 3 cm area on neck: *n* = 50)
**Hood 1:** 1 CFU of Bacillus Sp.	**Hood 3:** 1 CFU of Bacillus Sp.
**Hood 11:** Completely overgrown with a contaminant organism (> 100 CFU) (failed to identify) + 1 CFU of Bacillus Sp.	**Hood 4:** 1 CFU of Bacillus Sp.
**Hood 16**: 1 CFU of Bacillus Sp.	**Hood 11:** 1 CFU of Bacillus Sp.
	**Hood 23:** Swarming colony unquantifiable + Paenibaccillus Glucanolyticus

## Discussion

The presumption persists that early prosthetic joint infection originates from intraoperative contamination [5, 19]. Several studies have indicated that the majority of wound contamination during clean surgery, such as total joint arthroplasty, is caused by operating room personnel [5, 20]. However, it has also been shown that basic perioperative interventions can have a profound effect on infection rates. For example, a randomized clinical trial by Loftus et al. involving 236 adult patients, demonstrated that sustained improvements in basic perioperative preventive measures led to a substantial reduction in *S. Aureus* transmission and surgical site infections [21].

There is still a lack of consensus regarding the role of “positive pressure” SHS in preventing surgical infection [10, 13]. Regardless, SHS use remains widespread among the arthroplasty community. There are also no recommendations on the most appropriate method for hood donning and there are a variety of techniques and hood usage practices across different hospitals. Our results indicated a low glove contamination rate associated with SHS use when donned under laminar airflow with late fan activation. Furthermore, we did not observe a significant difference in sterility between these two commonly used donning techniques.

In a study examining surgical helmet and hood practices, Kang et al. found that late activation of the helmet’s fan system resulted in only minor levels of UV fluorescent powder dispersal compared to early activation. Additionally, they noted that adhesive wrist straps did not decrease powder dispersal when combined with late fan activation. Interestingly, the authors recommended that an unscrubbed member of staff apply all sterile hoods [22]. Similarly, Hanselman et al. found a significant difference in powder dispersal rates between late and early fan activation and recommended that gowning and gloving be completed before activation of the SHS fan [23]. Young et al. conducted a study analyzing fluorescent powder dispersal during mock surgical gowning. The fluorescent powder was applied to the surgeons’ hands to simulate skin shedding. Their study concluded that the positive pressure from SHS results in particle migration from the surgeon’s hands to the gown’s cuff, which necessitates a sealant tape around the inner glove [24]. The difference in particle contamination between a single-fan and a double-fan helmet design has also been researched. Vermeiren et al. found no difference between these two technologies but highlighted that glove-gown interface contamination was present in all tests with both systems [25]. It is important to note, however, that all of these studies caution that a link cannot be directly inferred between powder dispersal and PJI and that future microbiological studies are required.

Few microbiological studies have been performed in this area. Moores et al. examined the effect of laminar airflow and fan activation on particle counts and bacterial contamination rates using sterile hood systems. They found that having the fan switched on while scrubbing significantly increased bacterial contamination as well as particle counts by 3.7 times. They also demonstrated that all the exposure plates left open under laminar airflow were negative and therefore concluded that the most sterile technique of SHS donning is when it is performed under laminar airflow. The authors recommended only switching the fan on after the surgeon is completely gowned [26].

Bacillus species was the most frequently cultured organism in our study. This aligns with other studies that measure contamination of sterile materials. Bacillus Sp. are facultative anaerobes that are nutrient agnostic, allowing them to tolerate austere environments. They are also spore-forming, making them resistant to many chemical and heat sterilization processes [27, 28].

Our results also suggest the outer surface of the SHS screen and neckline area should not be presumed to be sterile. These hoods are marketed as sterile and often have removable plastic screens to allow intraoperative cleaning of the screen area. It is also common practice for some surgeons to adjust their helmets or fan settings intraoperatively. Similar findings were demonstrated by Kearns et al. who reported an SHS hood baseline contamination rate of 22% (22/102) at “time zero”, which increased to 47% (48/102) at the conclusion of a total joint arthroplasty procedure [29]. Other research by Singh et al. found that 80% of the SHSs used for 40 arthroplasty cases were contaminated with bacteria by the end of the procedure. In their study, the rate of contamination increased in 30-min intervals and was significantly higher when SHSs were used in non-laminar airflow theatres [30].

### Strengths and limitations

This is the first microbiological study to examine the difference between two commonly used SHS donning techniques. We also simulated typical gowning techniques in higher volumes than previous studies. It must be noted, however, that our study used simulated scrubbing which took place after working hours. This environment is likely more sterile than the day-to-day theatre environment and does not account for the usual staff traffic. Team members of equal height performed all the hood-donning trials. This may represent a source of variation that could contribute to contamination rates as has been shown in other studies involving assisted gowning procedures [18].

One limitation of the study is that not every contamination event leads to a postoperative complication. Thus, our study cannot directly imply a clinical increase in PJI or surgical site infection. Moreover, we only examined the glove contamination at time zero and did not take into account the potential for late contamination of bacteria from SHS to gloves or gowns. There was also only one brand of surgical hood system and gloves used in this study. This is relevant in our hospital but these results may not apply when using different technologies or materials.

## Conclusion

We found no statistically significant difference between the bacterial contamination rates of the operating surgeon’s gloves using two common techniques of SHS hood application. These trials were performed under laminar airflow with late fan activation. We advise that extreme care should be exercised when the operating surgeon assists donning a surgical colleague to negate the risk of inadvertent contamination. Importantly, based on our results, the SHS hood should not be presumed to be completely sterile after it has been applied and we recommend against adjusting it intraoperatively.

Further studies examining the clinical significance of these results are warranted. Research comparing different donning techniques using different brands of gloves and sterile hoods may further enlighten this area.

## Data Availability

All data used to support the findings of this research are present within the article.
